# Efficacy of HMJ-38, a new quinazolinone analogue, against the gemcitabine-resistant MIA-PaCa-2 pancreatic cancer cells

**DOI:** 10.37796/2211-8039.1423

**Published:** 2023-12-01

**Authors:** Mann-Jen Hour, Fuu-Jen Tsai, I-Lu Lai, Je-Wei Tsao, Jo-Hua Chiang, Yu-Jen Chiu, Hsing-Fang Lu, Yu-Ning Juan, Jai-Sing Yang, Shih-Chang Tsai

**Affiliations:** a School of Pharmacy, China Medical University, Taichung, 406040, Taiwan; b School of Chinese Medicine, College of Chinese Medicine, China Medical University, Taichung, 404333, Taiwan; c Human Genetics Center, Department of Medical Research, China Medical University Hospital, Taichung, 404327, Taiwan; d Department of Medical Genetics, China Medical University Hospital, Taichung, 404327, Taiwan; e Cell Therapy Center, China Medical University Hospital, Taichung, 404327, Taiwan; f Department of Nursing, Chung-Jen Junior College of Nursing, Health Sciences and Management, Chiayi, 62201, Taiwan; g Division of Plastic and Reconstructive Surgery, Department of Surgery, Taipei Veterans General Hospital, Taipei, 112201, Taiwan; h Department of Surgery, School of Medicine, National Yang Ming Chiao Tung University, Taipei, 112304, Taiwan; i Institute of Clinical Medicine, National Yang Ming Chiao Tung University, Taipei, 112304, Taiwan; j Department of Medical Research, China Medical University Hospital, China Medical University, Taichung, 404327, Taiwan; k Department of Biological Science and Technology, China Medical University, Taichung, 406040, Taiwan

**Keywords:** Gemcitabine-resistance MIA-PaCa-2 pancreatic cancer cells (MIA-GR100), HMJ-38, Epidermal growth factor receptor (EGFR), Autophagy, Apoptotic cell death

## Abstract

Gemcitabine is frequently utilized to treat pancreatic cancer. The purpose of our study was to create a gemcitabine-resistant MIA-PaCa-2 pancreatic cancer cell line (MIA-GR100) and to evaluate the anti-pancreatic cancer efficacy of HMJ-38, a new quinazolinone analogue. Compared to their parental counterparts, MIA-PaCa-2, established MIA-GR100 cells were less sensitive to gemcitabine. MIA-GR100 cell viability was not affected by 10, 50 and 100 nM gemcitabine concentrations. HMJ-38 reduced MIA-GR100 cell growth and induced autophagy and apoptosis. When stained with monodansylcadaverine (MDC), acridine orange (AO), and terminal deoxynucleotide transferase dUTP nick end labeling (TUNEL), MIA-GR100 cells shrunk, punctured their membranes, and produced autophagy vacuoles and apoptotic bodies. Combining chloroquine (CQ) and 3-methyladenine (3-MA) with HMJ-38 dramatically reduced cell viability, indicating that autophagy function as a cytoprotective mechanism. MIA-GR100 cells treated with both z-VAD-FMK and HMJ-38 were much more viable than those treated with HMJ-38 alone. HMJ-38 promotes apoptosis in MIA-GR100 cells by activating caspases. Epidermal growth factor receptor (EGFR) is one of HMJ-38’s principal targets, as determined *via in silico* target screening with network prediction. HMJ-38 also inhibited EGFR kinase activity and EGFR-associated signaling in MIA-GR100 cells. HMJ-38 may be an effective chemotherapeutic adjuvant for gemcitabine-resistant pancreatic cancer cells, in which it induces an antitumor response.

## 1. Introduction

Quinazolinone derivatives possess pharmacological actions including antibacterial activities [1], anti-angiogenesis activity [2], anti-microbial and anti-fungal activities [3], anti-malarial activity [4], analgesic activity [5], anti-tubercular activity [6], anticonvulsant activity [7], hypoglycemic activity [8] and anti-cancer activity [9,10]. New quinazolinone derivatives have been developed that have anti-cancer and anti-angiogenesis properties [2,9–11]. *In vitro* and *in vivo* studies have shown that HMJ-38 (2-(3′-methoxyphenyl)-6-pyrrolidinyl-4-quinazolinone) increases CDK1 activity and alters Bcl-2 phosphorylation in CAL 27 oral cancer cells and HL-60 leukemia cells [9,10]. Additionally, p53-regulated signaling also leads to DNA damage and death in HMJ-38-induced human umbilical vein endothelial cells (HUVECs) [11]. According to our preliminary findings, HMJ-38 may have cytotoxic effects on gemcitabine-resistant MIA-PaCa-2 pancreatic cancer cells (MIA-GR100), and its cytotoxicity could be attributed to autophagy and apoptosis.

Gemcitabine (2′,2′-Difluoro-2′-deoxycytidine) is the most commonly prescribed chemotherapeutic agent for pancreatic cancer [12]. An analog of deoxycytidine, gemcitabine is a pro-drug [13]. To become active, gemcitabine must be phosphorylated by deoxycytidine kinase into gemcitabine diphosphate (dFdCTP). dFdCTP inhibits DNA synthesis, leading to cell death in pancreatic cancer cells [14]. Gemcitabine has been reported to be effective in treating tumors such as pancreatic cancer, testicular cancer, breast cancer, non-small cell lung cancer, bladder cancer and ovarian cancer [15–17]. Several studies have demonstrated that gemcitabine resistance in pancreatic cancer cells is caused by intrinsic microenvironments, such as the overexpression of hypoxia inducible factor-1α (HIF-1α), nuclear factor κB (NF-κB) activity, matrix metalloproteinases (MMPs), heat-shock proteins (HSPs) and elevated PI3K/Akt kinase [18–23]. In contrast, epidermal growth factor receptors (EGFRs) contribute to the progression of osteosarcomas and non-small cell lung cancers, as well as the development of gemcitabine resistance [24,25]. Specifically, our study investigated whether HMJ-38 induced autophagic and apoptotic cell death in gemcitabine-resistant pancreatic cancer cells (MIA-GR100).

## 2. Materials and methods

### 2.1. Chemicals and reagents

Dr. Mann-Jen Hour synthesized a version of HMJ-38 (School of Pharmacy, China Medical University, Taichung, Taiwan) [2,9–11]. The following products were obtained from Sigma–Aldrich: acridine orange (AO), chloroquine (CQ), 3-methyladenine (3 MA), monodansylcadaverine (MDC), and 3-(4,5-dimethylthiazol-2-yl)-2,5-diphenyltetrazolium bromide (MTT). Fetal bovine serum (FBS), Glutamine, penicillin, RPMI1640 medium, streptomycin and trypsin EDTA were acquired from Thermo Fisher Scientific, Inc. The z-Val-Ala-Asp-fluoromethyl ketone (z-VAD-FMK; pan-caspases inhibitor) was purchased by R&D Systems (MN, USA).

### 2.2. Cell line establishes

Human pancreatic cancer MIA PaCa-2 from the Bioresource Collection and Research Center (BCRC Number: 60139) in Taiwan was purchased. By sub-culturing MIA-PaCa-2 cells and gradually increasing gemcitabine doses from 50 to 500 nM for six months, gemcitabine-resistant MIA-PaCa-2 pancreatic cancer cells (MIA-GR100) were generated [26,27]. MIR-GR100 and MIA PaCa-2 were cultured in MEM supplemented with 10% FBS, penicillin (100 U/mL), and streptomycin (100 μg/mL) at 37 °C with 5% CO_2_. For inhibitor assays, MIA-GR100 cells were pre-treated with chloroquine (CQ, 100 mM), 3-Methyladenine (3-MA, 10 mM), and z-Val-Ala-Asp-fluoromethyl ketone (zVAD-FMK, 10 mM) at 37C for 1 h, and then HMJ-38 cells were treated for the indicated durations.

### 2.3. Cell viability assay

The MTT (3-(4,5-dimethylthiazol-2-yl)-2,5-diphenyltetrazolium bromide) assay was used to evaluate cell viability. MIA-PaCa-2 and MIA-GR100 cells (2.5 × 10^5^ cells/well) were seeded on 24-well plates for 24 h. Cells were treated with gemcitabine (10, 50, 100, 1000 nM) or HMJ-38 (5, 10, 20 and 30 μM) for 24 h. In order to determine the viability of the cells, we performed the procedure described previously [28,29].

### 2.4. Real-time cell confluence

In this study, the IncuCyte S3 ZOOM System was employed to monitor the grown cells. MIA-GR100 cells (5 × 10^4^ cells/well) were plated in 96-well plates with or without HMJ-38 (10 μM) for 0–48 h. As previously mentioned, cells were visualized and photographed every 2 h [30,31].

### 2.5. Phase-contrast microscopy of morphological changes

The MIA-GR100 cells (2.5 × 10^5^ cells/well) were plated in 24-well plates with or without HMJ-38 (5, 10, and 20 μM) for 24 h. Using a phase-contrast microscope, the morphology of the cells was observed and photographed after 24 h [32,33].

### 2.6. Autophagy evaluation by acridine orange (AO) and monodansylcadaverine (MDC) staining

The MIA-GR100 cells (2.5 × 10^5^ cells/well) were seeded into 24-well plates with or without HMJ-38 (5, 10 and 20 μM) for 24 h. Cells were stained with acridine orange (AO) (1 μg/mL) or monodansylcadaverine (MDC) (50 μM) for period 20 min at room temperature. Stained cells were visualized using ImageXpress Micro Confocal High-Content Image System (Molecular Devices, LLC; magnification, × 400). Data analyzed using MetaXpress (version 5.3.0.4; Molecular Devices, LLC) to detect acidic vesicular organelles [31,34].

### 2.7. Apoptotic cell death evaluation by terminal deoxynucleotidyl transferase-mediated d-UTP nick end labeling (TUNEL)

The MIA-GR100 cells (2.5 × 10^5^ cells/well) were seeded into 24-well plates with or without HMJ-38 (5, 10 and 20 μM) for 24 h. DNA apoptosis was detected using TUNEL assays using the *In Situ* Cell Death Detection Kit according to the manufacturer’s instructions. Use the Counter NC3000 cytometer (ChemoMetec A/S) in accordance with the manufacturer’s instructions and the previously described procedure [26,35].

### 2.8. In silico studies of target screening and network analysis

The HMJ-38 structure was sketched using BIOVIA Draw. A total of 16,035 target proteins were screened for HMJ-38 using pharmacophore models in PharmaDB (BIOVIA Discovery Studio 2022 software; Dassault Systemes), with proteins showing a goodness-of-fit value greater than 0.6 being considered prospective HMJ-38 targets. To generate the relevant network of HMJ-38 targeted protein, human target genes were set as focus molecules and illustrated using a core analysis tool in IPA designated as focus molecules and visualized using IPA’s core analysis tool (IPA 2020; Qiagen Sciences, Inc.). A Fisher’s exact t-test (P < 0.05) indicated that the expected paths were statistically significant [28,36,37].

### 2.9. Molecular docking for EGFR

The PDB number for EGFR protein is 3UG2. The conformation of the isomers and tautomers of HMJ-38 and gefitinib was generated in order to establish different 3D structures with binding characteristics. The HMJ-38 and gefitinib were docked onto EGFR utilizing the CDOCKER technique included in the BIOVIA Discovery Studio 2022 program (Dassault Systems). The conformation of gefitinib and HMJ-38 with the highest CDOCK energy was selected as the most likely binding conformation [31,38].

### 2.10. EGFR kinase activity assay

As previously described [28,39], HMJ-38 exhibited EGFR kinase activity. The reaction mixture is comprised of 20 mM Hepes (pH 7.5), MgCl_2_ (10 mM), ethylene glycol tetraacetic acid (EGTA, 1 mM), polyoxyethylene Laural Ether (Brij35, 0.02%), bovine serum albumin (BSA, 0.02 mg/mL), Na_3_VO_4_ (0.1 mM), Dithiothreitol (DTT, 2 mM), dimethyl sulfoxide (DMSO, 1%), peptide substrate: poly [GLU:Tyr] (4:1) (5 mM) (GL Biochem, Shanghai), nicotinamide adenine dinucleotide (NADH, 0.5 mM). The process was initiated by the addition of EGFR kinase protein (0.5 mg/mL) and adenosine triphosphate (ATP, 0.2 mg/mL). The kinase reaction was conducted for 120 min at room temperature. The reactions are spotted onto ion exchange paper P81, and then washed thoroughly with 0.1% phosphoric acid. Using a microplate reader, luminous intensity was measured [28,39].

### 2.11. Statistical analysis

A total of three independent experiments were conducted for this study, the results are presented as the mean ± standard deviation (SD). To determine the differences between two and multiple groups, SPSS version 25.0 (IBM, Corp.) was used to perform a one-way analysis of difference, followed by Dunnett’s test and Tukey’s post-hoc test. A statistically significant difference was defined as one with a P value of 0.05 [40].

## 3. Results

### 3.1. The MIA-GR100 exhibits an increased resistance to gemcitabine

**Fig. 1:**
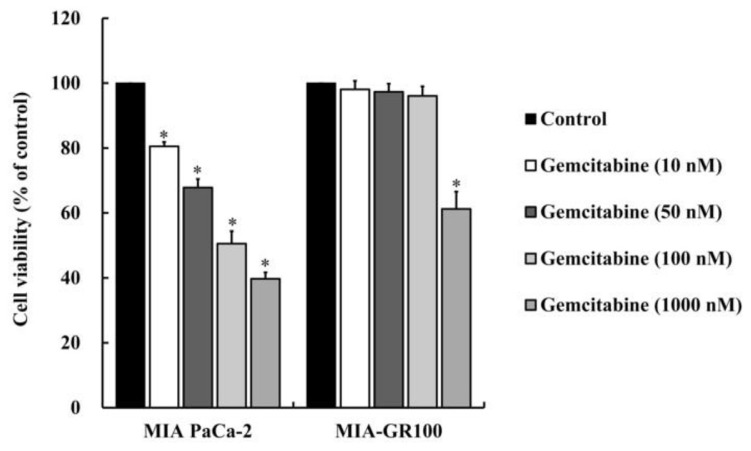
Effects of gemcitabine on the viability of MIA PaCa-2 and MIA-GR100 cells. MIA PaCa 2 and MIA-GR 100 cells (1x104 cells/well) were seeded into 96-well plates and treated with 0, 10, 50, 100 and 1000 nM of HMJ-38. Cytotoxicity was determined using the MTT assay. Three independent experiments are presented as the mean ± standard deviation (SD). Data were analyzed using one-way ANOVA followed by Dunnett’s post hoc test. *P < 0.05.

Gemcitabine resistance in pancreatic cancer remains a major clinical concern. The MIA-GR100 cell line has been established as resistant to gemcitabine. To investigate the effect of gemcitabine on the viability of human pancreatic MIA PaCa-2 cancer cells and gemcitabine resistance MIA PaCa-2 pancreatic cancer cells (MIA-GR100), we treated MIA PaCa-2 and MIA-GR100 cells with gemcitabine (10, 50, 100, and 1000 nM) for 24 h and then examined the viability of the cells using MTT analysis. As indicated in Fig. 1, MIA-GR100 was substantially more resistant to gemcitabine than MIA-PaCa-2.

### 3.2. HMJ-38 inhibits the viability of MIA-GR100 cells in vitro

**Fig. 2:**
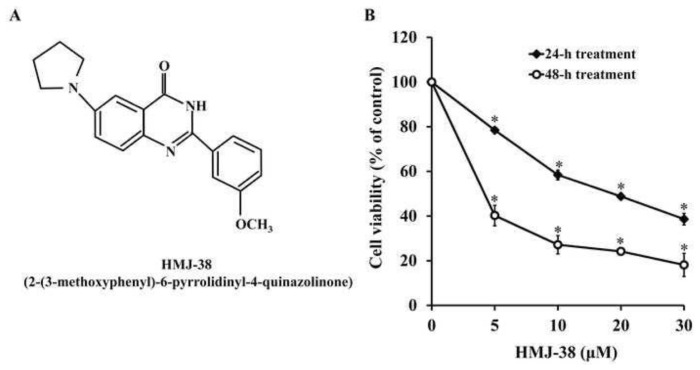
HMJ-38 reduces cell viability and exerts cytotoxicity in MIA-GR100 cells. (A) The chemical structure of HMJ-38. (B) HMJ-38 was added at 5, 10, 20, and 30 μM to MIA-GR100 cells. After 24 or 48 h of treatment, cell viability was assessed. Means ± SD are presented for three different experiments. The data were analyzed using one-way ANOVA followed by Dunnett’s post hoc test. *P < 0.05.

Fig. 2A displays the molecular structure of HMJ-38. MTT assay was used to determine the viability of HMJ-38 cells on MIA-GR100 cells. Compared to the control group, MTT result revealed a concentration- and time-dependent reduction in cell viability (Fig. 2B). Table 1 displays the half-maximal inhibitory concentrations (IC_50s_) for MIA PaCa-2 and MIA-GR100 cell lines treated with HMJ-38 for 24 and 48 h. HMJ-38 inhibited MIA PaCa-2 cells for 24 and 48 h with IC_50_ values of 25.83 ± 3.04 and 17.23 ± 2.11 μM, respectively. In addition, HMJ-38 inhibited MIA-GR100 cells for 24 and 48 h, with IC_50_ values of 18.57 ± 3.13 and 3.96 ± 1.01 μM, respectively.

### 3.3. Effects of HMJ-38 on MIA-GR100 cell growth using live-cell imaging

**Fig. 3:**
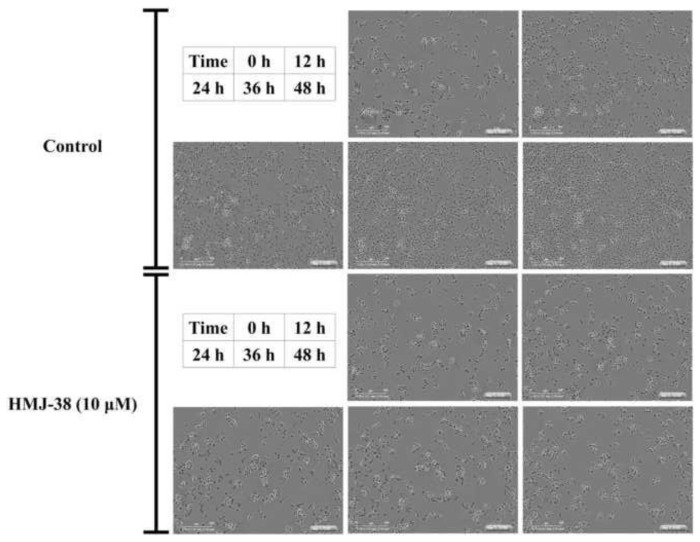
The IncuCyte image shows the effects of HMJ-38 on the confluence of MIA-GR100 cells. MIA-GR100 cells were observed and photographed at 0 h, 12 h, 24 h, 36 h, and 48 h following treatment with HMJ-38 (10 μM).

Confluence of cells might impact cell growth. The effects of HMJ-38 on cell confluence in control and HMJ-38 (10 μM)-treated cells were examined using IncuCyte S3 ZOOM live-cell imaging. The growth of cells was exhibited at 0, 12, 24, 36 and 48 h (Fig. 3). Contrary to HMJ-38-treated cells, the confluence of control cells increased with concentration and time (Fig. 3).

### 3.4. Cytotoxic effects of HMJ-38 on MIA-GR100 cell morphology

**Fig. 4:**
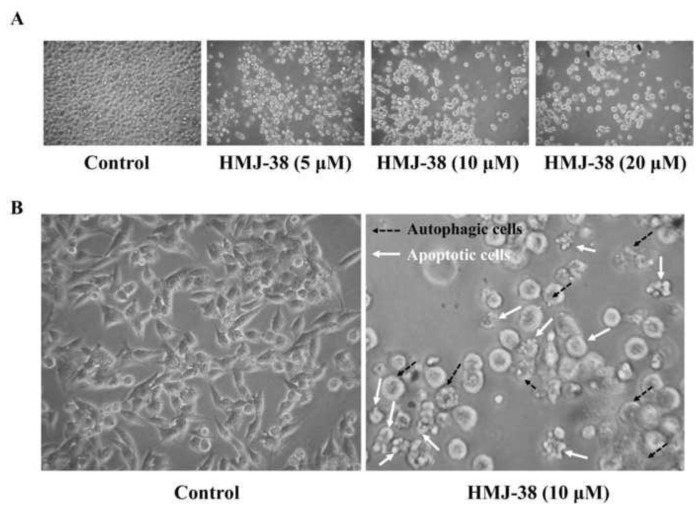
The morphological effects of HMJ-38 on MIA-GR100 cells. (A) In MIA-GR100 cells exposed to 0, 5, 10, and 20 μM HMJ-38, morphological alterations and cell death have been observed. (magnification, × 200). (B) Apoptotic (white dotted arrows) and autophagic (black solid arrows) characteristics were observed in HMJ-38 treated cells (magnification, × 400).

To investigate the effect of HMJ-38 on cell morphology, MIA-GR100 cells were treated for 24 h with HMJ-38 at concentrations of 5, 10 and 20 μM. HMJ-38-treated cells displayed concentration-dependent morphological changes, including rounding and shrinkage of cells (Fig. 4A). As shown in Fig. 4B, HMJ-38 (10 μM) treatment induces fragmentation and condensation of the nucleus, which is consistent with apoptosis or autophagy. The results of this study suggest that HMJ-38 could produce cytotoxic effects on MIA-GR100 cells that are associated with apoptosis and autophagy.

### 3.5. Induction of autophagy by HMJ-38 in MIA-GR100 cells

**Fig. 5:**
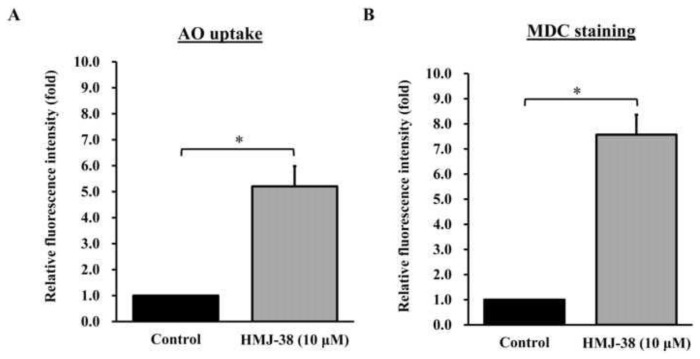
HMJ-38 induced autophagy in MIA-GR100 cells. There was a significant increase in the relative fluorescence intensity of AO uptake (A) and MDC staining (B) in MIA-GR100 cells treated with HMJ-38 compared to control cells. A mean ± SD is presented for three independent experiments. The data were analyzed using one-way ANOVA followed by Tukey’s post hoc test. *P < 0.05. AO, acridine orange; MDC, monodansylcadaverine.

To assess autophagy in cells, acridine orange (AO) and monodansylcadaverine (MDC) were used. MIA-GR100 cells were stained with AO or MDC 24 h after treatment with HMJ-38 (10 μM). There was a significant increase in the absorption of AO (Fig. 5A) and MDC (Fig. 5B) in HMJ-38-treated cells when compared to control cells. Our findings indicate that HMJ-38 treatment can trigger autophagy in MIA-GR100 cells.

### 3.6. HMJ-38 inhibits autophagy and promotes cell death in MIA-GR100 cells

**Fig. 6:**
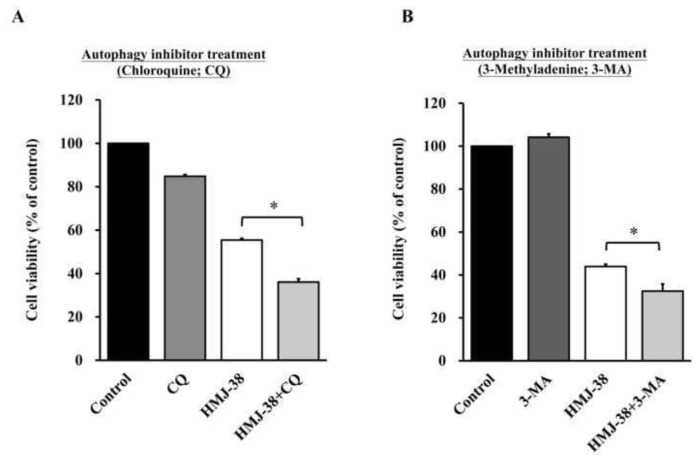
Inhibition of autophagy increases the cytotoxicity of MIA-GR100 cells treated with HMJ-38. The viability of cells after treatment with HMJ-38 and/or (A) CQ, (B) 3-MA. Data are presented as the mean ± SD from three independent experiments. The results were assessed using one-way ANOVA followed by Tukey’s post hoc test at a significance level of *P < 0.05.

Utilizing autophagy inhibitors such as chloroquine (CQ) and 3-methyladenine (3-MA), MIA-GR100 cells were treated with HMJ-38 in order to study the paradoxical effects of autophagy in tumor suppression and promotion [31,41]. MIA-GR100 cells treated with HMJ-38 and an autophagy inhibitor, CQ (Fig. 6A) or 3-MA (Fig. 6B), revealed a decrease in viable cells compared to HMJ-38-treated cells in Fig. 6. Based on these findings, HMJ-38-induced autophagy may have cytoprotective effects on MIA-GR100 cells.

### 3.7. Caspase-dependent apoptosis in MIA-GR100 cells is regulated by HMJ-38

**Fig. 7:**
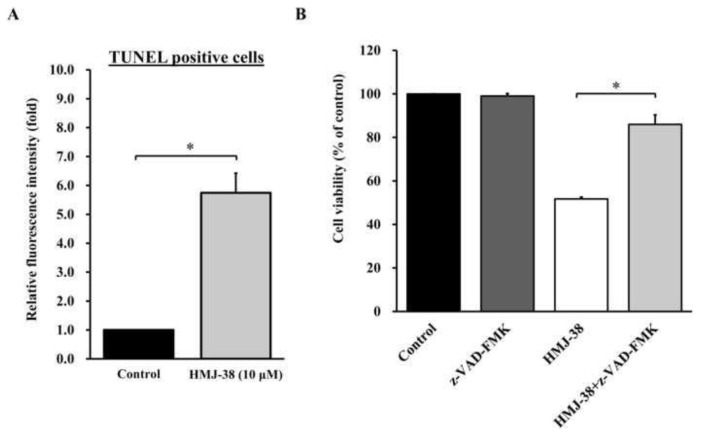
HMJ-38 activates the apoptotic signaling cascade in MIA-GR100 cells. (A) A TUNEL staining was used to determine the morphology of apoptotic cells induced by HMJ-38 compared to non-apoptotic cells treated with vehicle. Treatment with HMJ-38 increased the number of TUNEL-positive cells. (B) MIA-GR100 cells were treated with z-VAD-FMK (pan caspase inhibitor; 10 mM) for 1 h, followed by 24 h of treatment with 10 μM HMJ-38. The viability of the cells was measured after the cells were harvested. Data are presented as the mean ± SD from three independent experiments. The results were assessed using one-way ANOVA followed by Tukey’s post hoc test at a significance level of *P < 0.05.

To identify and quantify the number of apoptotic cells in MIA-GR100 cells, TUNEL assays were carried out on cells that were killed by HMJ-38. In comparison with control cells, HMJ-38 treatment significantly enhanced TUNEL-positive cells (Fig. 7A). Apoptosis was induced in MIA-GR100 cells by HMJ-38 with or without pan-caspases inhibitor (z-VAD-FMK) to determine the potential regulatory pathway. The cell viability of the HMJ-38-treated group was significantly lower than that of the other groups (Fig. 7B). HMJ-38-induced apoptosis in MIA-GR100 cells was caspase-dependent.

### 3.8. High throughput target screening predicts the target proteins for HMJ-38

**Fig. 8:**
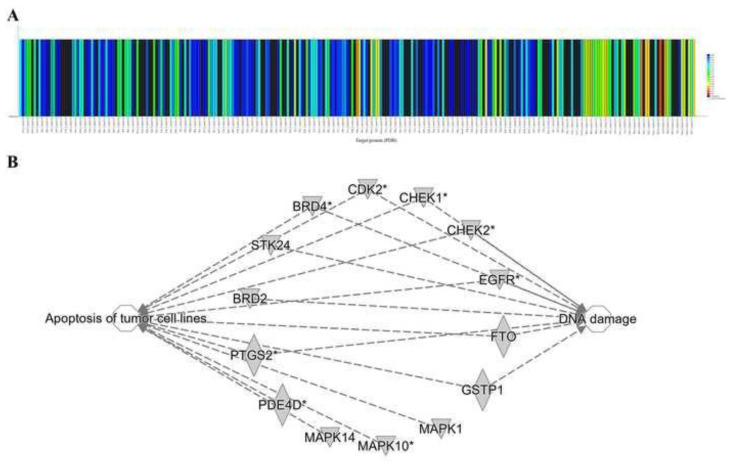
A high throughput target screening tool is utilized to detect HMJ-38 target proteins, and network is employed to predict the HMJ-38 pathway. (A) Using a high throughput platform for target screening, the binding patterns of HMJ-38-interacting proteins were analyzed. Red indicates a higher level of binding activity, whereas blue indicates a lower level of binding activity. (B) IPA core analysis of possible targets of HMJ-38 in apoptosis and DNA damage action.

**Fig. 9:**
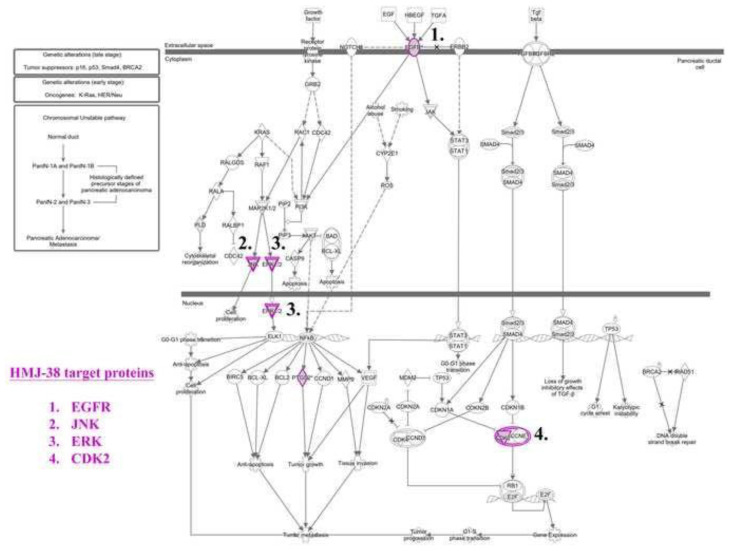
An analysis of the molecular signaling pathways targeted by HMJ-38 by IPA.

An interaction between HMJ-38 and other proteins involved in autophagic and apoptotic cell death has been suggested. Using a high throughput target screening platform, the HMJ-38 target proteins were predicted. As shown in Fig. 8A, proteins that interact with HMJ-38 have high binding activities. To further investigate the related-target proteins and their associated functions, the ingenuity pathway analysis database was utilized. Our findings indicate that HMJ-38 targets BRD4, CDK2, CHEK1/2, and EGFR, which have been implicated in the death of tumor cells and DNA damage pathways (Fig. 8B). As demonstrated in Fig. 9, the network was analyzed using ingenious pathway analysis (IPA). There are several receptors and pathways associated with the development of pancreatic ductal adenocarcinoma (PDAC), including the EGFR, the receptor protein tyrosine kinase, the NOTCH signaling system, the ERBB2, and the TGF bate pathway. A key function of EGFR is its participation in the JAK/STAT pathway, which is responsible for activating signaling cascades and transcribed genes. In Fig. 9, a network study of PDAC signaling pathways is illustrated. PDAC signaling is mediated by HMJ-38, which targets EGFR, JNK, ERK, and CDK2.

### 3.9. HMJ-38 targets EGFR and modifies EGFR kinase activity

**Fig. 10:**
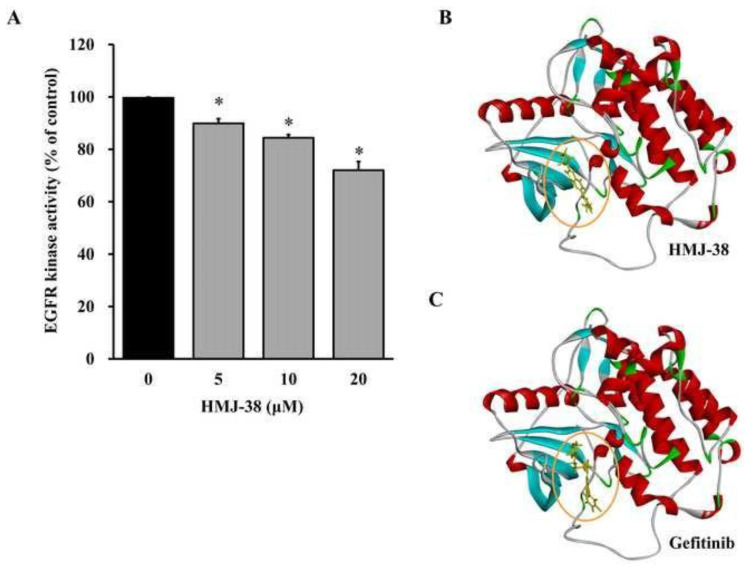
HMJ-38 interacts to EGFR kinase and suppresses its activity. (A) HMJ-38 inhibits EGFR kinase activity in a dose-dependent manner. Data are presented as the mean ± SD from three independent experiments. The results were assessed using one-way ANOVA followed by Tukey’s post hoc test at a significance level of *P < 0.05. (B) The crystal structure of EGFR reveals the position of the binding site for HMJ-38 and gefitinib.

It was hypothesized that HMJ-38 could target EGFR and impact the kinase activity of EGFR. With the HMJ-38 peptide, EGFR kinase testing was carried out. HMJ-38 displays a concentration-dependent suppression of EGFR activity (Fig. 10A). Following this, we will evaluate HMJ-38’s capacity to bind to EGFR. *In silico* analysis results on Fig. 10 demonstrates that the kinase domain of EGFR’s crystal structure interacts with HMJ-38 (Fig. 10B) and gefitinib (Fig. 10C). On the basis of these findings, HMJ-38 could target EGFR kinase and reduce its activity.

## 4. Discussion

There has been evidence that quinazolinone derivatives have antitumor effects on a variety of cancer cell lines [2,9–11,42–44]. HMJ-38 is a quinazolinone derivative that inhibits the polymerization of tubulin. *In vitro*, HMJ-38 interferes with the targeting position of tubulin, causing antimitotic, cytotoxic, and anticancer effects. Moreover, studies have shown that HMJ-38 induces cell cycle arrest in the G_2_/M phase and caspase-mediated cell death in HL-60 cells [10]. In HUVECs, HMJ-38 induces apoptosis through an extrinsic p53/ATM pathway [11]. The human oral cancer cell line CAL27 is susceptible to apoptosis induced by HMJ-38 through mitochondrial-mediated apoptosis and ER stress [9]. Studies have shown, however, that HMJ-38 has limited cytotoxicity in non-cancerous cells, such as the PBMC and human fibroblast cell lines, while exhibiting excellent resistance to cancerous cells. Hence, HMJ-38 is a potential oral anti-cancer drug deserving of further study.

This is the first study to examine the impact of HMJ-38 on pancreatic cancer cells exhibiting autophagy and apoptosis. According to IncuCyte ZOOM S3 live cell dynamic imaging, HMJ-38 reduced the cellular activity of MIA-RG100 after 48 h of exposure. MIA-RG100 is effectively inhibited by HMJ-38. Lysosomes play a crucial role in cellular clearance and energy production throughout the autophagy pathway.

However, it is unknown what role lysosomes play in HMJ-38-induced autophagy. Intriguingly, HMJ-38 was able to raise lysosomal acidity (Figs. 5 and 6) and increase autophagic lysosomal activity (Figs. 5 and 6), indicating that HMJ-38 activates lysosomes. A substantial reduction in the survival rate of MIA-RG100 cells was observed when the autophagy inhibitor was added to HMJ-38, indicating autophagy and indirectly confirming apoptosis. Our study found that autophagy inhibited apoptosis. Further, we discovered that the AKT-mTOR pathway regulates HMJ-38-induced lysosomal activation: HMJ-38 suppressed the protein levels of p-AKT and p-mTOR. The HMJ-38 protein promotes autophagy by de-phosphorylating AKT and mTOR, and it significantly up-regulates proteins located down-stream of the autophagy pathway (ATG-5, ATG-7, ATG-12, ATG-14, ATG-16, p62, and LC3) (Data not shown).

In order to understand how HMJ-38 causes apoptosis at the molecular level, proteins involved in the process were examined. Cytochrome *c* is released when the permeability of the inner mitochondrial membrane decreases, resulting in the formation of the apoptosome complex. The apoptosis-related factor (Apaf-1) also plays an important role in the formation of the apoptosome complex. Consequently, apoptosis is triggered. The recruitment of pro-caspase-9 to the apoptosome by the Apaf-1 complex activates caspase-9 auto-catalytically. By interacting with caspase-9, caspase-3, caspase-6, and caspase-7 increase apoptosis. HMJ-38 inhibits the synthesis of pro-caspase-3 and stimulates mitochondrial signaling pathways in MIA-RG100. The study sheds new light on the possibility that HMJ-38 is capable of inhibiting the growth of treatment-resistant pancreatic cancer MIA-RG100 cells (Fig. 7).

A nucleoside analog known as gemcitabine is integrated into the DNA strands of cancer cells during the S-phase [45,46]. In the process of DNA synthesis, it inhibits the production of deoxynucleotide triphosphates (dNTPs) by the enzyme ribonucleotide reductase [47,48]. Consequently, cancer cells are unable to duplicate their DNA and die as a result of apoptosis [47]. By inhibiting DNA production in cancer cells, gemcitabine prevents tumor development and spread [47,49]. EGFR gene mutations are responsible for the response of NSCLC to gefitinib [47,49]. Gefitinib inhibits the activity of the EGFR protein, which regulates cell growth and division through its cell surface receptor. NSCLC cells multiply and divide uncontrollably when the EGFR proteins are altered [47,49]. The action of gefitinib is inhibited as a result of its ability to slow or stop the proliferation of cancer cells, as a result of its ability to inhibit mutant EGFR proteins. HMJ-38 inhibits EGFR activity in a concentration-dependent manner (Fig. 10A). Molecular docking of EGFR *in silico* revealed that the crystal structure’s kinase domain interacts with HMJ-38 (Fig. 10B) and/or gefitinib (Fig. 10C). HMJ-38 has been proposed as a potential EGFR inhibitor. This agent inhibits EGFR activity and EGFR-AKT-mTOR signaling, thereby inhibiting the proliferation of pancreatic cancer cells resistant to gemcitabine (MIA-GR100).

In conclusion, this study demonstrated that HMJ-38 induces autophagy and apoptosis in Gemcitabine-resistant MIA-RG100 pancreatic cancer cells. HMJ-38 exhibited significant cytotoxicity in MIA-RG100 cells, induced autophagy through EGFR-AKT-mTOR signaling and induced apoptosis through mitochondrial signaling. A proposed molecular mechanism of HMJ-38 activity sheds fresh light on its anti-gemcitabine-resistant cancer cells properties. HMJ-38 has the potential to be employed in pancreatic cancer treatment or in combination with other medications. HMJ-38 may contribute to the fight against gemcitabine-resistant MIA-PaCa-2 pancreatic cancer in the future.

## Figures and Tables

**Table 1:** The half-maximal inhibitory concentrations (IC50s; μM) for the treatment of MIA PaCa-2 and MIA-GR100 cells with HMJ-38 after 24 and 48 h.

Cell lines	24-h	48-h
MIA-GR100	18.57 ± 3.13 (μM)	3.96 ± 1.01 (μM)
MIA PaCa-2	25.83 ± 3.04 (μM)	17.23 ± 2.11 (μM)
